# An Assessment of Maternal Health Issues in Two Villages in the Eastern Cape Province of South Africa

**DOI:** 10.3390/ijerph110909871

**Published:** 2014-09-22

**Authors:** Amanda Siruma, Diana Hornby, Sunitha Srinivas

**Affiliations:** 1 Division of Pharmacy Practice, Faculty of Pharmacy, Rhodes University, Grahamstown 6140, South Africa; E-Mail: tafmanda@gmail.com; 2 Community Engagement Office, Rhodes University, Grahamstown 6140, South Africa; E-Mail: d.hornby@ru.ac.za

**Keywords:** maternal health, South Africa, service delivery, antenatal care, adolescent pregnancy

## Abstract

The fifth Millennium Development Goal of improving maternal health was placed on the international agenda and endorsed by global leaders at the Millennium Summit held in 2000. The aim of this baseline study was to conduct a situational analysis of key maternal health issues in two rural Eastern Cape villages in South Africa: Glenmore and Ndwayana. Ten focus group discussions were conducted with village leaders, community health workers and three different women self-help groups from Glenmore and Ndwayana, with five to eight voluntary participants in each focus group discussion. One of the themes highlighted was inadequate service delivery of ambulance services, which frequently failed to timeously reach expectant mothers in urgent need of transportation to a referral hospital. Adolescent pregnancy was highlighted as the maternal health issue of most concern to the community participants. In this context, a consensus was reached to design and implement an educational intervention to address adolescent pregnancy, which will form the focus of the next phase of this project.

## 1. Introduction

The year 2000 marked the launch of eight time-bound Millennium Development Goals (MDGs) at the Millennium Summit, and MDG 5 is to improve maternal health [1]. Focus on the severity of maternal mortality as the main cause of death in women of reproductive age led to the United Nations General Assembly prominently situating maternal health as part of the international development agenda at this Millennium Summit [2]. MDG 5 identifies two targets for assessing progress in improving maternal health: reducing the maternal mortality ratio (MMR) by three-quarters between 1990 and 2015 and achieving universal access to reproductive health by 2015. According to the MDG Report for 2013, progress has been made to reduce maternal deaths globally, as the MMR has declined by 47% over the past two decades, from 400 maternal deaths per 100,000 live births in 1990 to 210 in 2010 [3].The MDG Report for 2012 highlighted the proportion of maternal deaths occurring in developing countries as accounting for 85% of the global burden, with 56% occurring in Sub-Saharan Africa and 29% in South Asia [4]. Maternal mortality exhibits the greatest disparity between the developed and developing world [5]. Of great concern is that, although some regions have shown progress since 1990 in reducing maternal deaths, MMRs in Sub-Saharan Africa have remained very high, with little evidence of improvement in the past 20 years [6].

According to global classification by the World Bank, South Africa is a middle income country [7]. Regrettably, South Africa features amongst the Sub-Saharan countries with a high and increasing maternal mortality rate [8]. According to the World Health Organization (WHO) Health Profile for South Africa, an MMR of 400 maternal deaths per 100,000 live births is estimated for the country [9]. In response, focusing on decreasing maternal and child mortality has been identified as one of the key strategic outputs by the National Department of Health [10]. Maternal healthcare has become one of the priority reproductive health issues that have been identified as requiring urgent attention in South Africa by the National Maternity Guidelines Committee in its manual for clinics, community health centers and district hospitals [11].

The aim of this baseline study was to investigate the facilitating and constraining factors with regard to maternal health issues in the two rural Eastern Cape village communities based on feedback from the community participants and to identify the perception of community participants regarding the maternal health issue of most concern to them.

## 2. Methods

Ethical clearance to conduct the study was obtained from the Rhodes University Faculty of Pharmacy’s Ethics Committee. The study setting was two Eastern Cape rural communities: Glenmore and Ndwayana. The Angus Gillis Foundation (AGF), a local non-governmental organization and a partner in this study, facilitated access to the study setting and introduced the researcher to the prospective participants. After obtaining their informed consent to participate voluntarily in the baseline study, focus group discussions (FGDs) were used as a data collection tool. The use of FGDs was preferred over quantitative methods, as it is useful in investigating unquantifiable aspects regarding maternal health issues in the respective communities [12].

### 2.1. Data Collection 

Each FGD had five to eight participants to facilitate group interaction. Permission to use a tape recorder was requested, and written informed consent was sought from all FGD participants. Five FGDs were conducted with the village leaders, women self-help groups and community health workers (CHWs) in each of the two villages. Among the women self-help groups, three FGDs were conducted in each community: one each for elderly women (grandmothers), older women (experienced mothers) and young women (pregnant women and/or mothers with new-born babies).

To ensure uniformity, all FGDs were moderated by the principle investigator along with the same positive health champion as the interpreter. “Positive health champion” is a term used by AGF and pertains to a member of the community who is trained and mentored by AGF to assist in the facilitation of health workshops and liaison between the community and their primary healthcare center (PHC).

All FGDs were conducted in IsiXhosa, which is one of South Africa’s eleven official languages and a commonly used language in the Eastern Cape province. The discussions took place in a venue that was convenient for the community participants. Prior to initiation of each FGD, a brief explanation of maternal health was given to ensure that participants understood the topic of discussion. Participants were encouraged to speak freely while giving each person the opportunity to speak and elaborate on an issue. Each FGD was approximately 45 minutes long, and the data collection process was carried out from April to July 2012. Thereafter, recordings of the FGDs were transcribed verbatim and then translated to English by language experts from the School of African Languages at Rhodes University.

### 2.2. Data Analysis

Thematic analysis was used in this study to portray the prevalent themes highlighted in the narratives given by the participants. To facilitate this process, data analysis was managed using the Nvivo^®^ 10 data analysis computer software program. Transcripts from all ten FGDs were imported into the software and then categorized into themes. Navigation within the software facilitated extraction and linking of similarly-related themes, a process referred to as coding. Thereafter, coded information was moved to folders, which are called nodes. All coded data was compared within each node and further analyzed to see if new themes emerged. Each set of emerging themes was pooled with similar information within other nodes and then organized under a new resultant node.

## 3. Results 

Overall, 67 women and nine men participated in the FGDs. Table 1 shows the details of the FGD participants from Glenmore and Ndwayana.

**Table 1:** Number of focus group discussions (FGD) participants from Glenmore and Ndwayana.

FDG Participant Groups	Village	Number of Participants	Number of Male Participants	Number of Female Participants
Village leaders	Glenmore	7	3	4
	Ndwayana	7	6	1
Community health workers	Glenmore	8	0	8
	Ndwayana	7	0	7
Elderly women (grandmothers)	Glenmore	7	0	7
	Ndwayana	8	0	8
Older women (experienced)	Glenmore	8	0	8
	Ndwayana	8	0	8
Young women (pregnant women and/or mothers with new-born babies)	Glenmore	8	0	8
	Ndwayana	8	0	8
Total number of participants	Glenmore	38	3	35
	Ndwayana	38	6	32

Similar information was obtained from FGDs conducted in both villages. Based on the FGDs conducted with the women’s self-help groups, CHWs and village leaders, the following themes emerged.

### 3.1. Challenges with Antenatal Care

From all ten FGDs, it was gathered that most pregnant women access the PHCs for antenatal care services, but there are no resources to conduct delivery when an expectant mother is in labor or in case an obstetric emergency occurs. As a result, expectant mothers are referred to a public sector hospital located approximately forty-five kilometers away. This information was emphasized in the four FGDs conducted with the older women who are experienced mothers and young women who were either pregnant women and/or mothers with new-born babies. FGD participants mentioned that in most instances, healthcare professionals at the PHC would not call an ambulance, but would give them a referral letter, and the maternal patient would have to make transport arrangements to get to the referral hospital. This proved to be a challenge for maternal patients who did not have money for transport to access the referral hospital. Another issue pointed out was the lack of equipment, which also led to the need for a referral. Some participants expressed the following sentiments:

Experienced mother from Glenmore: “I think I have got a problem with maternal health services. They are using the old method of checking people; like fiddling with their hands. It is rare to find them using machines. The reason I left this clinic is they didn’t see the problem, so I decided to leave the clinic, because they just said I have water in the stomach, there’s only one child, when instead I was carrying twins. So I told myself it is not the right clinic for me. I will not say they need more training, because they are well trained; they need to have more equipment at the clinic”.

Young mother from Glenmore: “We haven’t ever seen someone deliver a child here, because there are no services for that; some of us go to Nompumelelo or some of us just go to Grahamstown”.

### 3.2. Ambulance Services 

Most FGD participants in all ten FGDs highlighted that there were challenges with accessing ambulance services for women in labor. Participants stated that ambulances would take eight hours to arrive or sometimes would not come at all. A few participants indicated that there were regulations set by the National Department of Health prohibiting pregnant women from giving birth at home. The challenge with ambulance services arriving on time thus often led to the occurrence of emergency home births. Elderly women who were not health professionals, but had experience in conducting home births, would assist in such circumstances, but were not allowed to cut the umbilical cord. As an alternative, they would have to keep the “afterbirth” in a plastic bag, while they waited for a qualified healthcare professional to come and cut the umbilical cord. The ambulance and health care professionals regularly arrived late, and FGD participants expressed concern over the risks associated with this. Similar sentiments were expressed by the following participants:

Experienced mother from Ndwayana: “Ambulances take long to arrive; if you call an ambulance at about 2 o’clock you get that ambulance at about 11 p.m.”.

Elderly woman from Glenmore: “They say the ambulances are few and there are lots of patients to attend to. We are told the ambulances are still in East London (a nearby city) and busy, so you can imagine it is a big town with lots of rural areas depending on ambulances from there, like King Williamstown, so they are advised by the nurses to try and get money to hire transport just in case”.

### 3.3. The Unavailability of Healthcare Professionals during Weekends

Glenmore and Ndwayana villages each have one PHC, which is operational only during weekdays. It was further expressed in eight of the ten FGDs that the unavailability of healthcare professionals during weekends is a risk factor for expectant mothers who may experience complications during this time and need urgent medical attention and/or a referral to a hospital. An example is the occurrence of emergency home births, as mentioned in the previous paragraph. Similar sentiments were expressed by the following participants:

Young mother from Glenmore: “There are no nurses during the weekend, and if something were to happen to a pregnant woman, it would not be easy to get medical help. We also need to have a doctor coming more frequently to the clinic instead of once a month”.

Male village leader from Glenmore: “What I have noticed is that there is no care at the clinic after 4:30 and during the weekends, so maybe if the clinic would open all the days of the week and at night it would be helpful”.

### 3.4. Reproductive Health Services at PHCs

FGD participants expressed that although there is accessibility to bio-medical interventions, such as contraceptive injections, pills and condoms, there is a need to incorporate workshops on HIV/AIDS (human immunodeficiency virus/acquired immunodeficiency syndrome) issues and adolescent pregnancy as part of the reproductive health services package. Some participants mentioned the need to promote abstinence as a choice of contraception, as this would help in the prevention of adolescent pregnancy and against possible contraction of HIV and STIs (sexually transmitted infections). These sentiments were expressed by participants in nine out of ten FGDs. Related sentiments were articulated by the following participants:

Experienced mother from Ndwayana: “There is nothing much that we get from the clinic, rather than the injection and the condoms and pills. There are no induction workshops about issues related to HIV, teenage pregnancy, STDs, and so on”.

Elderly woman who is a grandmother from Glenmore: “We are told if you have birth at an early age, you are susceptible to cancer. I don’t know what can make those children be on family planning or the way we have grown in the olden days of abstaining as a better option of preventing teenage pregnancy”.

### 3.5. High Rate of Adolescent Pregnancy

The need to promote the prevention of adolescent pregnancy due to their high occurrence in the community was highlighted in nine out of ten FGDs. The FGDs with the elderly women who are grandmothers from both villages expressed that they were concerned about the underdeveloped bone structure of the young girls in their community, which increased the risk of complications during child birth. From the four FGDs with elderly women and experienced mothers, there was concern that often, as mothers of the pregnant adolescent girls, they would have to bear the responsibility of bringing up the newborn infant instead of the adolescent mother. Reasons highlighted included the adolescent mother needing to return to school or the neglect of the newborn infant by the adolescent mother, which most FGDs participants viewed as intentional. Other participants highlighted the concern they had with regard to pregnant adolescents having their educational development interrupted, and most times, they would not return to school. Another sentiment that was highlighted was the possibility of young girls becoming pregnant as a way of obtaining the child support grant (CSG). There was general concern that becoming pregnant at such a young age had consequences that went beyond the perceived advantages of the CSG, which was reported as R 300 per month at the time of the study. Related sentiments were expressed by the following participants:

Experienced mother from Glenmore: “Some said it is difficult to have pocket money since their parents are not working, therefore an easy way out for them to have some money is through child support grant. I want to believe it is around R 300, therefore you can imagine if someone has about three children, she is standing on about plus-minus R 900 per month”.

Elderly mother who is a grandmother from Glenmore: “By getting pregnant so early, our children are abusing us, as they expect us to take care of their children. As the girl’s parent, we now have to do everything for the child, including taking them to the clinic for immunizations”.

Experienced mother from Ndwayana: “There is a problem with this early pregnancy amongst girls, as they are affecting their education, and it seems there is no way of stopping it when I hear them talk. They should go back to the olden days, as there were a group of older women who checked if someone had touched a girl and if so, they would bring the child in front of a committee. When the child says who it is, a group of younger woman would go out singing bad things about that person to punish them for entering their daughter at an early stage without permission”.

### 3.6. Need for Educational Intervention on Adolescent Pregnancy

There was a general consensus by participants in nine of the ten FGDs that the design and implementation of an educational intervention focusing on adolescent pregnancy would form the basis of the next phase of this study. Most FGD participants highlighted that adolescent pregnancy posed a threat to the empowerment of young girls by limiting their physical and educational development. In this context, such interventions targeting adolescent girls were viewed by FGD participants as important, not only in improving reproductive and maternal health outcomes, but also in optimizing the overall transition of adolescent girls to adulthood. Some participants expressed the following sentiments:

Experienced mother from Glenmore: “These things are being taught at the clinic; every time they (adolescent girls) go, there is someone to sit you down from this table to that table. They (adolescent girls) are being pressed about prevention of pregnancy. Maybe if somebody from outside would come and talk to them, maybe they would listen”.

Elderly woman who is a grandmother from Glenmore: “If we can be helped in teaching our children how important their bodies are so they can acknowledge and appreciate their bodies and they can be taught the ways to abstain from everything that will spoil those bodies until they are of age to have children or it is the right time, which is when they are married”.

Male village leader from Glenmore: “They say there is no remedy, but my advice is that if we can organize some training and raise awareness, because if the rate of pregnancy is high, there are no condoms that are used by them”.

### 3.7. Hypertension in Pregnancy

Hypertension in pregnancy was indicated as a maternal health issue of main concern in the FGD conducted with the village leaders in Ndwayana. Although one participant misconstrued that a pregnant woman was considered to be ill, he associated hypertension in pregnancy as a potential health condition that could occur during pregnancy. 

Male village leader from Ndwayana: “When a woman is pregnant, she is considered to be sick, and the things that can affect her are things, such as high blood (hypertension)”.

Female village leader from Ndwayana: “I would like to know if you can take high blood (hypertension) in pregnancy (as a maternal health issue of most concern), because if you have this, usually at a later stage, you can get diabetes. So, the community can be taught about hypertension in pregnancy”.

### 3.8. Role of Community Health Workers

Participants from all ten FGDs highlighted the importance of CHWs as a liaison between the community and the PHC. The responsibilities of the CHWs ranged from home visits to promote use of antenatal care services at PHC and campaigns for immunizations.

Community health worker from Glenmore: “As health workers, if we see a pregnant woman in the community, we do a home visit and advise her to go to the clinic and get a checkup. They (pregnant women) must come at the earlier stage to get a checkup, so that if there is anything that needs to be treated, this is done early”.

Male village leader from Glenmore: “Information that helps to promote maternal health is given to pregnant women by volunteers (community health workers) who are at the clinic”.

Elderly woman who is a grandmother from Ndwayana: “As the community health workers are field workers, they advise pregnant women (by going) house to house, to come to the clinic, so that they can get a checkup”.

## 4. Discussion 

The Eastern Cape is the second poorest province in South Africa and has the highest level of unemployment, with reduced access to health services, impacting negatively on the population [13,14]. In addition, public health systems are greatly challenged by the shortage of healthcare professionals [15]. Glenmore and Ndwayana are located north of the Great Fish River in the former Ciskei homeland, which is now part of rural Eastern Cape [16]. Both villages face issues of poor infrastructure and limited services, including transport to the nearest cities, which provide access to public sector health institutions upon which the majority of the local population is dependent.

Lack of infrastructural resources and unavailability of staff during weekends in marginalized communities, such as Glenmore and Ndwayana, pose a challenge towards improving maternal health outcomes. In spite of this, according to the South Africa Health Profile for 2010, South Africa has made progress concerning women who received skilled care at childbirth living in rural and urban areas between 1990–1999 and 2000–2008 from 84% to 91%, respectively [9]. In December 2011, the neighboring Free State province’s Department of Health issued 48 ambulance vehicles, with 18 of these specifically designated for maternity/obstetrics patients. This initiative assisted in the implementation of more efficient and timely inter-facility transport of maternal patients [17]. In addition, according to a statement issued by the Eastern Cape Department of Health on January 12, 2012, 60 ambulances have been added to the fleet of ambulances serving the province [18]. One of the goals of these efforts is to speed up the delivery of ambulance services to the Eastern Cape community in order to help reduce maternal mortality and morbidity. The Nelson Mandela metropolitan area of the Eastern Cape has benefited from this program, resulting in improvement of waiting times for ambulance services [19]. WHO’s *Monitoring the Building Blocks of Health Systems: A Handbook of Indicators and their Measurement Strategies* highlights that “comprehensiveness, accessibility, coverage, continuity, quality, person-centeredness and coordination” are key characteristics to good service delivery. Furthermore, enhanced service delivery, including access to services, is an expected result of increased inputs in a health system [20]. From the FGD discussions, it is apparent that there are still challenges with regard to ambulance services reaching marginalized areas, such as Glenmore and Ndwayana timeously, and this is a constraining factor when addressing maternal health issues.

Notwithstanding these challenges, the principles of PHC are still very applicable, highlighting a critical need for the accountability of all stakeholders concerned [21]. In 1997, South Africa drafted the *White Paper on Public Service Transformation*, which provides a guideline for health providers in public service delivery and serves to improve the value of delivering services that meet the basic needs of all South Africans. The aforementioned document further calls upon policy-makers to identify and make available services that redirect resources to communities that are underserved [22]. A set of transformation priorities were established, which set out to transform public service delivery into a more community-orientated approach, in which the community is aware of their health rights at every level of health services they acquire. These were termed the eight Batho Pele principles [23]. One of the principles of Batho Pele emphasizes that access to decent public services is the rightful expectation of all citizens, especially those previously disadvantaged [23]. Other national documents, such as the *Primary Health Care Package for South Africa—A Set of Norms and Standards*, also facilitate the opportunity for South Africa’s population to know what quality of primary healthcare services they should expect to receive [24].

The role of CHWs in primary healthcare is relatively well recognized in the Glenmore and Ndwayana communities. CHWs play an active role in promoting antenatal services for pregnant women in the two villages as a facilitating factor towards addressing maternal health issues. In May 2011, the National Department of Health announced a new policy on the re-engineering of the Primary Health Care system and the overhaul of the health system. The new policy is committed to four strategic outputs that the health sector must achieve, with the second output focusing on decreasing maternal and child mortality [25]. The document has emphasized the role of CHWs as central to the PHC initiative with a focus on maternal, child and women’s health. Among the above-mentioned duties, each CHW is also assigned to be responsible for health promotion and prevention at the household and community level [26].

Hypertension in pregnancy was highlighted as the maternal health issue of most concern following feedback from one FGD conducted with the village leaders in Ndwayana. According to the Department of Health’s Saving Mothers report on confidential inquiries into maternal deaths in South Africa, complications of hypertension in pregnancy are reported amongst adolescent girls as forming an unreasonably large group who die from pre-eclampsia and eclampsia [27].

Data concerning reproductive health services and the high rate of adolescent pregnancy, which was highlighted in all ten FGDs conducted, confirms the relevance of including reproductive health as a target for improving maternal health. The right to universal access to reproductive health, which had not initially been stated under the Millennium Declaration, was incorporated as a clear target in 2007 and became effective on 1 January 2008. Indicators to monitor progress in achieving this developmental target are the contraceptive prevalence rate, adolescent birth rate, antenatal care coverage and the unmet need for family planning [28]. Statistics from the South African Statistical Fact Sheet for 2010 show that the percentage of unmet need for family planning for Africa is 24.3%, whilst no data was recorded for South Africa [29].

In nine out of the ten FGDs, most participants felt there was a need for the young girls in the community to be aware of abstinence as a choice of contraception, which should be included in reproductive health campaigns instead of solely promoting condom use. According to the South Africa Health profile for 2011, the contraceptive prevalence rate for Africa is 24%, whilst that for South Africa is 56% [9]. Although South Africa has a higher contraceptive prevalence rate than the rest of Africa, the promotion of educational interventions that highlight abstinence is important as a non-biomedical approach to contraception. Apart from the provision of health services at healthcare facilities, the dissemination of reproductive health information that does not focus solely on the use of biomedical contraceptives, particularly for adolescent girls, is an important aspect of reproductive health rights [30]. In 2004, the National Department of Health adopted the ABC approach, which highlights “A” for abstinence, “B” for being faithful and “C” for condom use [31]. The ABC approach uses interventions that are specific to different population groups. While all aspects of the ABC approach are deemed equally important, for adolescents, the emphasis is on abstinence and delaying sexual debut until marriage [32].

Adolescent pregnancy poses higher risks, including the risk of anemia, eclampsia and higher vulnerability to other causes of maternal death. Achieving sexual and reproductive health facilitates the opportunity for individuals to develop in other aspects, such as education. Moreover, babies born to adolescent mothers are also at risk of neonatal death. Failure to improve sexual and reproductive health has adverse outcomes, as it relates to adolescent pregnancies, which expose affected individuals to the risk of STI and HIV infection in the long term [33].

South Africa has a formal social security system, which assists vulnerable segments of the population from the complete brunt of poverty. Such assistance is in the form of social grants; examples are the disability grant, older person grant and CSG, among others [34]. Commentary has been made questioning the sustainability of the social grants, which have an allocation of 3.5% of South Africa’s GDP [35]. Currently, the CSG is R 300 a month per child. Individuals who are eligible for the CSG are primary care givers who earn less than R 34,800 per year if single or R 69,600 if married [36]. Notions of some adolescent girls becoming pregnant as a way to obtain the CSG were highlighted during the FGDs. The provision of the CSG has raised wide spread public views that associate this social grant with increased fertility, particularly within the adolescent population. Acquiring short-term perceived benefits of the CSG and lack of comprehension of the actual costs that are involved in bringing up a child are thought to be the reason for this [37]. Paradoxically, despite the availability of the CSG in South Africa and the popular notion that young women are becoming pregnant to receive this grant, one study reported that only 2.69% of grant recipients were mothers between the ages of 15 and 19 years [38]. Other studies on the CSG and its relationship with increased adolescent pregnancies have asserted that the phenomenon of adolescent pregnancy predates the introduction of the CSG [39,40].

Data from nine of the ten FGDs conducted in this study highlighted compelling sentiments from community participants with regard to the need to address adolescent pregnancy through an educational program in the community. WHO and the United Nations Population Fund (UNFPA) both highlight prevention of adolescent pregnancy as an important and cost-effective intervention, which contributes towards attaining MDG 5 [33]. South African policy guidelines on teenage pregnancy, such as *Teenage Pregnancy in South Africa—With a Specific Focus on School-Going Learners*, highlight the negative impact of adolescent pregnancy on increasing maternal deaths and emphasize the need for active involvement of all stakeholders as crucial to addressing adolescent pregnancy [41]. Improved access to sexual and reproductive health, which includes access to services and information, is a mainstay for supporting the overall health of communities, in particular that of women. The achievement of MDG 5 and other health-related MDGs is strongly reinforced by the progress that can be made on sexual and reproductive health. Health promotion and community participation, which facilitates the opportunity for subsequent community empowerment, are a cost-effective approach to improving maternal and reproductive health outcomes, particularly in remote underserved communities [42]. In this context, an educational intervention to raise awareness of the health risks associated with adolescent pregnancy will be designed accordingly and implemented in the next phase of this research.

## 5. Conclusions

The FDG participants in this study identified the proactive role of CHWs in promoting antenatal care at the PHCs, which is a facilitating factor in improving maternal health outcomes. The factors potentially restraining positive maternal health outcomes were challenges with irregular ambulance services and a lack of comprehensive equipment for antenatal care in the PHCs. Improvements in the aforementioned factors are more dependent on the involvement of key legislative stakeholders in mobilizing resources at the provincial level. Adolescent pregnancy was highlighted as the maternal health issue of most concern to the community participants in both villages. In this context, to facilitate active community participation, the design and implementation of an educational intervention to address adolescent pregnancy will form the focus of the next phase of this project.

## References

[1] (2005). Health in the Millennium Development Goals.

[2] United Nations The Millennium Development Goals Report 2001. http://www.un.org/millenniumgoals/pdf/MDG%20Report%202010%20En%20r15%20-low%20res%2020100615%20-.pdf.

[3] United Nations The Millennium Development Goals Report 2013. http://www.un.org/en/development/desa/publications/mdg-report-2013.html.

[4] United Nations The Millennium Development Goals Report 2012. http://www.un.org/en/development/desa/publications/mdg-report-2012.html.

[5] (2007). Maternal Mortality in 2005: Estimates Developed by WHO, UNICEF, and UNFPA.

[6] Hill K., Thomas K., Zahr C.A., Walker N., Say L., Inoue M., Suzuki E. (2007). Estimates of maternal mortality worldwide between 1990 and 2005: An assessment of available data. Lancet.

[7] (2010). List of Economies.

[8] United Nations Children’s Fund Countdown to 2015 Report: Maternal and Newborn and Child Survival 2012. http://countdown2015mnch.org/documents/2012/country_countsmall.pdf.

[9] World Health Organization South Africa Health Profile. http://www.doh.gov.za/docs/stats/2011/stats2011who.pdf.

[10] (2007). Saving Mothers: Third Report on Confidential Inquiries into Maternal Deaths in South Africa, 2002–2004.

[11] (2007). National Maternity Guidelines Committee at the Department of Health. A Manual for Clinics, Community Health Centres and District Hospital.

[12] Morgan D.L. (1997). Focus Groups as Qualitative Research.

[13] Human Sciences Research Council Fact Sheet: Poverty in South Africa 2004. http://www.sarpn.org.za/documents/d0000990.

[14] (2010). Eastern Cape Province Spatial Development Plan.

[15] Province of the Eastern Cape Annual Performance Plan, Department of Health 2009–2011. http://www.ecdoh.gov.za/uploads/files/260308124845.pdf.

[16] Punt C. (2005). A Profile of the Eastern Cape Province: Demographics, Poverty, Inequality and Unemployment. http://www.elsenburg.com/provide/documents/BP2005_1_2%20Demographics%20EC.pdf.

[17] Schoon M.G. (2013). Impact of inter-facility transport on maternal mortality in the Free State Province. S. Afr. Med. J..

[18] Government Statement Eastern Cape, South Africa 2012. http://www.info.gov.za/speech/DynamicAction?pageid=461&sid=32427&tid=91132.

[19] Reducing Maternal and Child Mortality through Strengthening Primary Health Care in South Africa. Improving EMS Waiting Times in Eastern Cape. www.rmchsa.org/wpcontent/uploads/2014/03/DCST_Case_StudyImproving_EMS_Waiting_Times_ECape_Final.pdf.

[20] World Health Organisation Monitoring the Building Blocks of Health Systems: A Handbook of Indicators and Their Measurement Strategies. http://www.who.int/healthinfo/systems/WHO_MBHSS_2010_section1_web.pdf?ua=1.

[21] What is Primary Health Care? University of Cape Town: Faculty of Health Sciences, 2012. http://www.primaryhealthcare.uct.ac.za/approach/whatis/whatis.htm.

[22] Wagner A., Graves A., Reiss S., LeCates R., Zhang F., Ross-Degnan D. (2011). Access to care and medicines, burden of health care expenditures, and risk protection: Results from the World Health Survey. Health Policy.

[23] (2007). Batho Pele Principles—Putting People First.

[24] (2000). Primary Health Care Package for South Africa—A Set of Norms and Standards.

[25] National Department of Health Human Resources Health South Africa Strategy for the Health Sector: 2012/13–2016/17. http://www.doh.gov.za/docs/stratdocs/2011/hrh_strategy.pdf.

[26] National Department of Health Re-Engineering of Primary Health Care in South Africa. http://www.ahp.org.za/news-detail/229/the-reengineering-of-primary-healthcare-in-south-africa#sthash.s8YIVZSm.dpuf.

[27] (2012). Saving Mothers: Report on Confidential Inquiries into Maternal Deaths in South Africa 2008–2010.

[28] World Health Organization Millennium Development Goals (MDGs). http://www.who.int/mediacentre/factsheets/fs290/en/.

[29] World Health Organization South African Statistical Sheet, 2010. www.who.int/countries/zaf.

[30] African Union The African Charter on Human and People’s Rights, 2003. www.africaunion.org/root/au/documents/treaties/treaties.htm.

[31] Speech by Minister of Health at the 2004 Abstinence Walk Fundraising Send off Party. http://www.doh.gov.za/show.php?id=845.

[32] (2003). United States President’s Emergency Plan for AIDS Relief. http://www.pepfar.gov/.

[33] United Nations Population Fund (2012). Early Marriages, Adolescent and Young Pregnancies, Sixty-Fifth World Health Assembly. http://www.who.int/gb/ebwha/pdf_files/WHA65/A65_13-en.pdf.

[34] Government of the Republic of South Africa. Department of Social Development: Grants 2013. http://www.dsd.gov.za/.

[35] Sustainability of the South African Welfare State, 2013. http://www.sanlam.co.za/wps/wcm/connect/sanlam_en/sanlam/investor+relations/economic+information/economic+commentary/the+sustainability+of+the+south+african+welfare+state.

[36] Government of the Republic of South Africa South African Government Services Child Support Grant 2013. http://www.services.gov.za/services/content/Home/ServicesForPeople/Socialbenefits/childsupportgrant/en_ZA.

[37] Case A., Hosegood V., Lund V. (2004). The reach and impact of Child Support Grants: Evidence from KwaZulu‐Natal. Develop. S. Afr..

[38] Makiwane M., Desmond C., Richter L., Udjo E. (2006). Is the Child Support Grant Associated with an Increase in Teenage Fertility in South Africa? Evidence from National Surveys and Administrative Data.

[39] Woodward L.J., Harwood L., Fergusson D.M. (2001). Teenage pregnancy: Cause for concern. N. Z. Med. J..

[40] Goldblatt B., Solange R., Hall K. (2005). Implementation of the Child Support Grant: A Study of Four Provinces and Recommendations for Improved Service Delivery.

[41] Panday S., Makiwane M., Ranchod C., Lestoalo T. (2009). Teenage Pregnancy in South Africa—With a Specific Focus on School-Going Learners.

[42] World Health Organization Global Experience of Community Health Workers for Delivery of Health Related Millennium Development Goals. http://www.who.int/workforcealliance/knowledge/publications/alliance/Global_CHW_web.pdf.

